# Insertion Sequence (IS)-Excision Enhancer (IEE)-Mediated IS Excision from the *lacZ* Gene Restores the Lactose Utilization Defect of Shiga Toxin-Producing Escherichia coli O121:H19 Strains and Is Responsible for Their Delayed Lactose Utilization Phenotype

**DOI:** 10.1128/aem.00760-22

**Published:** 2022-08-01

**Authors:** Keiji Nakamura, Kazuko Seto, Junko Isobe, Itsuki Taniguchi, Yasuhiro Gotoh, Tetsuya Hayashi

**Affiliations:** a Department of Bacteriology, Graduate School of Medical Sciences, Kyushu University, Fukuoka, Japan; b Division of Planning, Osaka Institute of Public Health, Osaka, Japan; c Department of Bacteriology, Toyama Institute of Health, Toyama, Japan; University of Tartu

**Keywords:** IS-excision enhancer (IEE), Shiga toxin-producing *Escherichia coli* (STEC), delayed lactose utilization, insertion sequence (IS)

## Abstract

Lactose utilization is one of the general biochemical characteristics of Escherichia coli, and the *lac* operon is responsible for this phenotype, which can be detected on lactose-containing media, such as MacConkey agar, after 24 h of incubation. However, some Shiga toxin-producing E. coli (STEC) O121:H19 strains exhibit an unusual phenotype called delayed lactose utilization (DLU), in which lactose utilization can be detected after 48 h of cultivation but not after only 24 h of cultivation. Insertion of an insertion sequence (IS), IS*600*, into the *lacZ* gene appears to be responsible for the DLU phenotype, and exposure to lactose has been reported to be necessary to observe this phenotype, but the mechanism underlying these phenomena remains to be elucidated. Here, we performed detailed analyses of the lactose utilization abilities of a set of O121:H19 strains and their mutants and found that IS-excision enhancer (IEE)-mediated excision of IS*600* reactivates the *lacZ* gene and that the selective proliferation of IS-cured subclones in lactose-supplemented culture medium is responsible for the expression of the DLU phenotype. In addition, we analyzed the patterns of IS insertion into the *lacZ* and *iee* genes in the global O121:H19 population and revealed that while there are O121:H19 strains or lineage/sublineages that contain the IS insertion into *iee* or intact *lacZ* and thus do not show the DLU phenotype, most currently circulating O121:H19 strains contain IS*600*-inserted *lacZ* and intact *iee* and thus exhibit this phenotype.

**IMPORTANCE** Insertion sequences (ISs) can modulate gene expression by gene inactivation or activation. While phenotypic changes due to IS insertion/transposition are frequently observed, gene reactivation by precise or simple IS excision rarely occurs. In this study, we show that IS*600* is excised from the *lacZ* gene by IS-excision enhancer (IEE) during the cultivation of Shiga toxin-producing Escherichia coli (STEC) O121:H19 strains that show an unusual phenotype called delayed lactose utilization (DLU). This excision rescued their lactose utilization defect, and the subsequent selective proliferation of IS-cured subclones in lactose-containing medium resulted in the expression of the DLU phenotype. As we also show that most currently circulating O121:H19 strains exhibit this phenotype, this study not only provides information helpful for the isolation and identification of O121:H19 STEC but also offers novel insights into the roles of IS and IEE in the generation of phenotypic variation in bacterial populations.

## INTRODUCTION

Insertion sequences (ISs) are small transposable elements (typically 0.7 to 2.5 kb in size) that encode a transposase (TPase) and are usually flanked by terminal inverted repeats (IRs) (1). More than 4,000 ISs were deposited in the ISFinder database, and they are classified into 32 families based on several features, such as the sequences of their TPases and IRs (1, 2). IS transposition induces a variety of genome rearrangements, including deletion, inversion, and duplication (3, 4), and generates small structural polymorphisms (5), which have strong impacts on the genome diversification and evolution of bacteria (6).

Another important aspect of the impact of IS insertion/transposition on their bacterial hosts is the modulation of gene expression by gene inactivation or activation (7, 8). While phenotypic changes due to IS insertion are frequently observed in many bacterial species, there have been very few reports on gene reactivation by IS excision because precise or simple excision of IS elements rarely occurs in bacterial cells (9, 10). However, the IS-excision enhancer (IEE) identified in Shiga toxin-producing E. coli (STEC) strains promotes the excision of IS*3* family members and generates various types of genomic alterations, including simple IS excision and deletion of IS-flanking regions (11). The *iee* gene is encoded by large integrative elements, SpLE1 in the O157:H7 strain Sakai and SpLE1-like elements in other STEC serotypes (O26:H11, O111:H8, O103:H2, O145:H28, and O121:H19) (11–13). IS*3* family members, such as IS*629*, IS*1203*, and IS*600*, have been amplified in these STEC serotypes (12–16), and notable phenotypic and genotypic variations have been generated within these STECs by IS transposition and IEE-mediated IS excision (5, 15, 17, 18).

Lactose fermentation is one of the general biochemical characteristics of coliform bacteria belonging to four genera of *Enterobacteriaceae* (*Citrobacter*, Enterobacter, Escherichia, and Klebsiella) (19). E. coli utilizes lactose by hydrolyzing the β-1,4 glycosidic bond by β-galactosidase encoded by the *lacZ* gene, which forms the *lac* operon along with *lacY* and *lacA* encoding a permease and a transacetylase, respectively (20). While lactose utilization, which can be detected on lactose-containing agar plates, such as MacConkey agar (referred to as MAC), after 24 h of incubation is a trait often used to identify E. coli, it has recently been reported that some STEC O121 strains exhibit an unusual phenotype called delayed lactose utilization (DLU) (21). In DLU strains, lactose utilization was observed on MAC after 48 h of incubation but not after only 24 h of incubation, and exposure to lactose was necessary to observe this phenotype. As a copy of IS*600* was inserted into *lacZ* in the DLU strains, this IS insertion appears to be related to the DLU phenotype, but the mechanism underlying this phenomenon has not been previously elucidated.

In this study, we performed detailed analyses of the lactose utilization ability of a set of O121:H19 strains and their mutants under several culture conditions and revealed that IEE-mediated IS*600* excision reactivates the *lacZ* gene and that the selective proliferation of subclones that contain the IS-cured *lacZ* gene in lactose-containing medium is responsible for the expression of the DLU phenotype. In addition, we analyzed the patterns of IS insertion into *lacZ* and *iee* in the global O121H19 population and showed that while most currently circulating O121:H19 clones exhibit the DLU phenotype, some O121:H19 strains or sublineages contain IS insertion into *iee* and thus do not show this phenotype.

## RESULTS

### IS*600* excision from the *lacZ* gene during cultivation.

**FIG 1:**
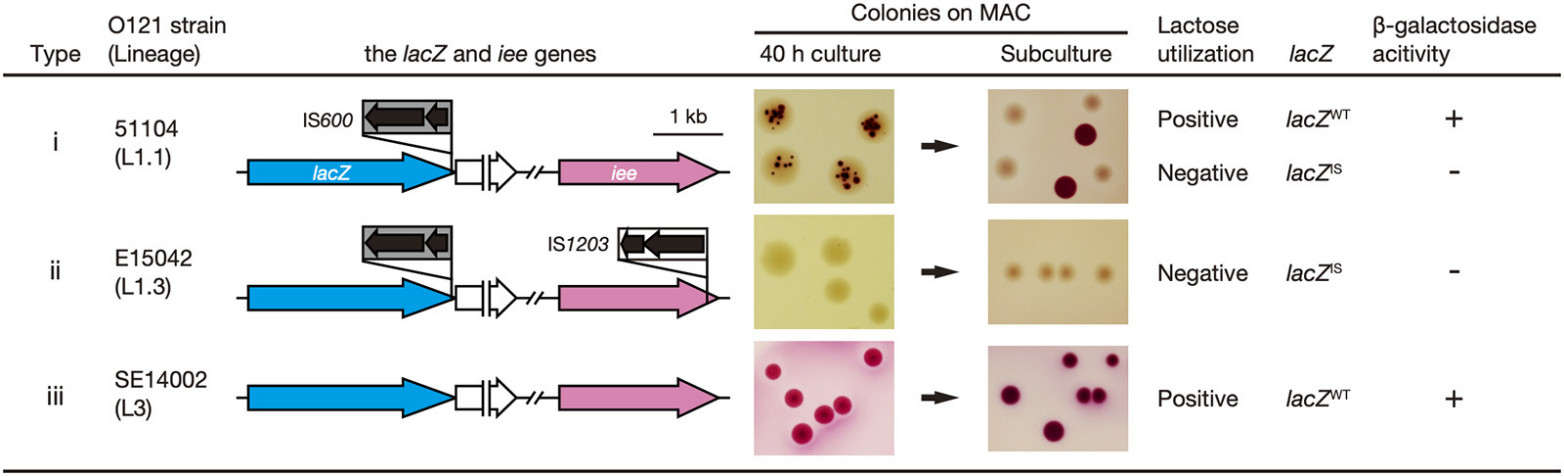
IS insertion into the *lacZ* and *iee* genes and lactose utilization abilities of three O121:H19 strains. In the left part, IS insertions into *lacZ* and *iee* in three O121:H19 strains are shown. These strains belong to different lineages or sublineages, as shown in parentheses (see Fig. 5A and the Results section for details). In the middle part, colonies of the O121:H19 strains cultured for 40 h on MAC and colonies reisolated from their 16 h subculture on MAC are shown. In the right part, the results of PCR analysis to detect the IS-inserted *lacZ* gene (*lacZ*^IS^) and the β-galactosidase activity of each strain are shown (Fig. S1 and S2 for more details of the results).

As the IEE promotes the excision of IS elements belonging to the IS*3* family (11) and O121:H19 strains contain an SpLE1-like element that encodes the *iee* gene (13), we hypothesized that the DLU phenotype is caused by IEE-mediated IS*600* excision from *lacZ*. By reinspecting the genome sequences of O121:H19 strains (13) available in our laboratory, we identified three types of strains that differ in the intactness of *lacZ* and *iee*: (i) IS*600*-inserted *lacZ* (referred to as *lacZ*^IS^) and wild-type *iee* (*iee*^WT^), (ii) *lacZ*^IS^ and IS*1203*-inserted *iee* (*iee*^IS^), and (iii) wild-type *lacZ* (*lacZ*^WT^) and *iee*^WT^ (Fig. 1). We selected three representative strains, one of each type (the genome sequences of these strains have been finished [13]) (Table 1) and cultured them for 40 h on MAC. Strains E15042 and SE14002 (types ii and iii, respectively) clearly showed a negative and positive lactose-fermentation phenotype, respectively. However, in strain 51104 (type i), red microcolonies were formed in translucent colonies, indicating the emergence of lactose-fermenting subclones during colony growth (Fig. 1). By subculturing the colony of strain 51104 onto MAC, we obtained both translucent and red colonies after 16 h of incubation. PCR analysis of the genomic DNA extracted from these colonies revealed that IS*600* was not present in the *lacZ* gene in red colonies but was present in that of translucent colonies (Fig. 1 and Fig. S1). As IS*600* insertion occurred very close to the 3′ end of *lacZ* (Fig. S2A), it is not clear whether the IS insertion inactivated β-galactosidase. We therefore examined the β-galactosidase activity of each colony by the 5-bromo-4-chloro-3-indolyl-β-d-galactopyranoside (X-gal) assay and found that *lacZ*^IS^ colonies did not show β-galactosidase activity (Fig. 1 and Fig. S2B). These results indicated that in the *iee*^WT^ strain, the excision of IS*600* from *lacZ* occurred during cultivation on MAC, resulting in the emergence of β-galactosidase (*lacZ*)-reactivated subclones.

**TABLE 1:** Strains and plasmids used for the analysis of lactose utilization

Strain or plasmid	Description^*a*^	Source or reference (Accession No.)
Strains		
51104	O121:H19; *lacZ*^IS^, *iee*^WT^	(13) (AP024471-AP024472)
51104Δ*iee*	51104 derivative; *lacZ*^IS^, *iee*::Cm^R^	This study
E15042	O121:H19; *lacZ*^IS^, *iee*^IS^	(13) (AP024478-AP024479)
SE14002	O121:H19; *lacZ*^WT^, *iee*^WT^	(13) (AP024473-AP024474)
K-12	Wild type; *lacZ*^WT^, *iee*^WT^	(38) (NC_000913)
K-12_*lacZ*^IS^	K-12 derivative; *lacZ*^IS^	This study
K-12_*lacZ*^IS^:pBR	K-12_*lacZ*^IS^ derivative carrying pBR322; Ap^R^, Tc^R^	This study
K-12_*lacZ*^IS^:p*iee*	K-12_*lacZ*^IS^ derivative carrying pBR-*iee*; Tc^R^	This study
Plasmids		
pBR322	Cloning vector; Ap^R^, Tc^R^	TaKaRa (J01749)
pBR-*iee*	pBR322 derivative with ECs1305 (*iee*) and its flanking region; Tc^R^	This study

a *lacZ*^WT^, wild-type *lacZ*; *lacZ*^IS^, IS*600*-inserted *lacZ*; *iee*^WT^, wild-type *iee*, *iee*^IS^, IS*1203*-inserted *iee*; Cm^R^, chloramphenicol resistance; Ap^R^, ampicillin resistance; Tc^R^, tetracycline resistance.

### Involvement of IEE in IS*600* excision from *lacZ*.

**FIG 2:**
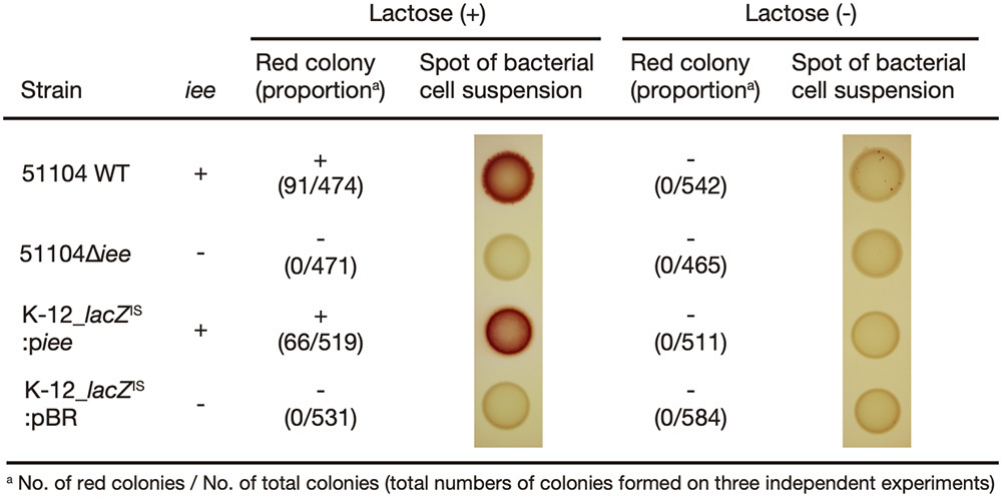
Involvement of IEE in the emergence of lactose-fermenting subclones during cultivation and the effect of lactose. The proportion of lactose-fermenting subclones in the colonies of each strain cultured on MacConkey agar base plates supplemented with or without 1% lactose for 40 h at 37°C was determined by suspending 5 colonies in LB, inoculating the suspensions on MacConkey agar base supplemented with 1% lactose (MAC plate), and counting red colonies after 16 h of incubation at 37°C. For spot analyses, cell suspensions (approximately 1.3 × 10^7^ CFU) were spotted onto MAC plates and grown for 16 h at 37°C, and representative patterns are shown for each strain and culture condition.

The finding that red microcolonies representing *lacZ*-reactivated subclones were not formed on MAC in strain E15042 (type ii; carrying *iee*^IS^) (Fig. 1) suggested that IEE is responsible for the excision of IS*600* from *lacZ*. To verify this hypothesis, we constructed an *iee* deletion mutant of strain 51104 (51104Δ*iee*) and two K-12 derivatives that carried the same *lacZ*^IS^ gene as that in strain 51104 and either a plasmid encoding the *iee* gene (K-12_*lacZ*^IS^:p*iee*) or an empty plasmid vector (K-12_*lacZ*^IS^:pBR) (Table 1), and we examined the emergence of subclones carrying the IS*600*-excised *lacZ* gene after 40 h of cultivation on MAC (Fig. 2). Red (lactose-fermenting) colonies emerged from the wild-type 51104 strain and K-12_*lacZ*^IS^:p*iee* but not from 51104Δ*iee* and K-12_*lacZ*^IS^:pBR. In the two *iee*-negative mutants, lactose-fermenting colonies were not detected even in the spots of the bacterial suspension (1.3 × 10^7^ CFU/spot). These results indicated that the excision of IS*600* from *lacZ* is mediated by IEE.

### Increase in the subpopulation carrying IS*600*-excised *lacZ* in the stationary phase during cultivation in lactose-supplemented medium.

When the wild-type 51104 strain and the K-12_*lacZ*^IS^:p*iee* strain were cultured for 40 h on nonlactose-supplemented MAC (MacConkey agar base) plates, no lactose-fermenting colonies were obtained, although small numbers of red colonies were detected in the spot of the 51104 suspension (Fig. 2). IEE-mediated IS excision requires an active IS TPase (11); however, the TPase gene in the IS*600* copy inserted into *lacZ* is transcribed in the direction opposite to that of the *lac* operon (Fig. 1), and thus, it is likely that the increased IS*600* TPase expression by the induction of the *lac* operon is not involved in the enhancement of IS*600* excision. To verify this hypothesis, we examined the effect of isopropyl-β-d-thiogalactopyranoside (IPTG) supplementation on the emergence of subclones carrying IS*600*-excised *lacZ* (Fig. S3). K-12_*lacZ*^IS^:p*iee* cultured in lysogeny broth (LB) supplemented with IPTG yielded a small number of lactose-fermenting subclones, but the numbers of such subclones were similar to or even lower than those in LB medium not supplemented with IPTG. This result suggests that the induction of the *lac* operon has no detectable impacts on the IS*600* excision frequency.

**FIG 3:**
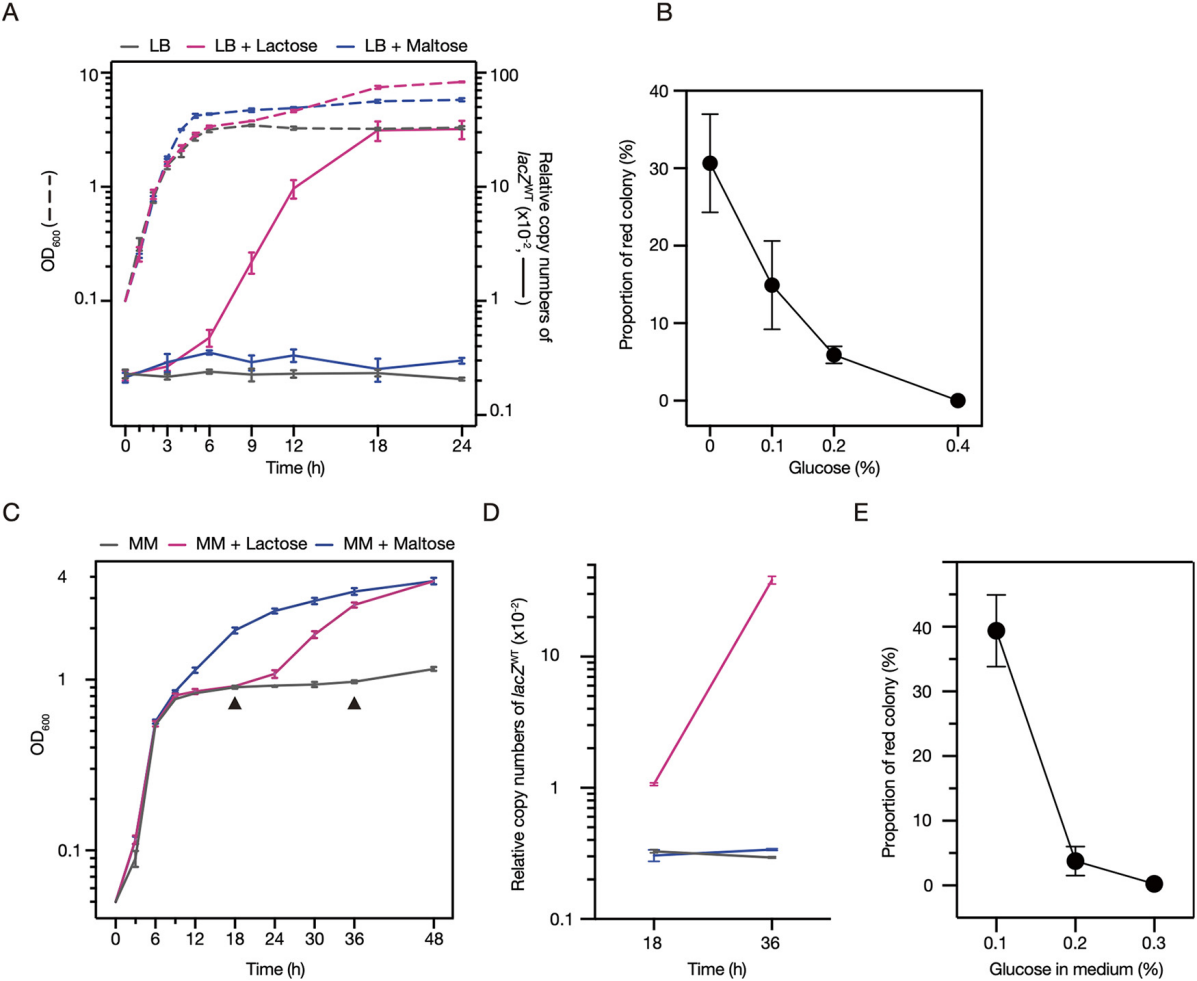
The emergence of lactose-fermenting subclones during the culture of K-12_*lacZ*^IS^:p*iee* in LB and the chemically defined minimal medium supplemented with various carbon sources. (A) Growth of K-12_*lacZ*^IS^:p*iee* cultured in LB or LB supplemented with lactose (1%) or maltose (1%) and the changes in the copy number of IS-excised *lacZ* during cultivation are shown by dotted lines. The relative copy numbers of IS-excised *lacZ* in the total cellular DNA were determined at each time point by calculating the ratio of the copy number of IS-excised *lacZ* relative to the copy number of the *lacY* gene and are shown by solid lines. (B) The proportions of lactose-fermenting subclones in the 18-h cultures of K-12_*lacZ*^IS^:p*iee* grown in LB supplemented with 1% lactose and different concentrations of glucose. The proportions were calculated by dividing the number of red colonies by the number of total colonies on MAC plates. The mean values with standard deviations of biological triplicates are shown. (C and D) Growth curves of K-12_*lacZ*^IS^:p*iee* cultured in MM and MM supplemented with 1% lactose or 1% maltose (C) and the changes in the copy number of IS-excised *lacZ* during cultivation (D). The relative copy numbers of IS-excised *lacZ* in the total cellular DNA were determined at 18 h and 36 h (indicated by arrowheads in Fig. 3C) as described for panel A. (E) The proportions of lactose-fermenting subclones in the 18-h cultures of K-12_*lacZ*^IS^:p*iee* grown in MM supplemented with 1% lactose and different concentrations of glucose. The proportions were calculated as described for panel B. All OD_600_ values and relative copy numbers in this figure are presented as the mean value with standard deviations of biological triplicates and are shown on a logarithmic scale.

We next analyzed the growth of the K-12_*lacZ*^IS^:p*iee* strain in LB supplemented with either lactose or maltose (1% each; wt/vol), the latter of which is also utilized by E. coli as a carbon source (22) (dotted lines in Fig. 3A). The addition of maltose increased the bacterial density in the stationary phase. In contrast, while the strain cultured in the presence of lactose grew similarly to the control culture (supplemented with neither lactose nor maltose) until 6 h after inoculation, it exhibited further growth between 9 h and 18 h after inoculation. This result suggested the possibility that this second growth phase represents the growth of subclones carrying IS*600*-excised *lacZ* by utilizing lactose. To verify this hypothesis, we monitored the temporal change in the proportion of these subclones in the cultures grown in each medium by determining the copy number of *lacZ*^WT^ relative to that of the *lacY* gene (solid lines in Fig. 3A). The relative copy number of *lacZ*^WT^ was maintained at a low level in the strain cultured in LB and maltose-supplemented LB (2~3 × 10^−3^ copies) throughout the 24-h cultivation. In contrast, in the strain cultured in lactose-supplemented LB, the relative copy number of *lacZ*^WT^ started to increase at 6 h and reached approximately 3 × 10^−1^ copies at 18 h. When various amounts of glucose were added to the medium, the proportion of *lacZ*^WT^-carrying subclones decreased in a glucose concentration-dependent manner (Fig. 3B), indicating that lactose utilization was inhibited by carbon catabolite repression (23).

We further performed similar analyses of K-12_*lacZ*^IS^:p*iee* using a chemically defined minimal medium (MM, see Materials and Methods for its composition) (Fig. 3C to E). These analyses also clearly showed the maltose utilization and delayed lactose utilization of the strain (Fig. 3C), the increase in the relative copy number of *lacZ*^WT^ by delayed lactose utilization (Fig. 3D), and the inhibition of lactose utilization by carbon catabolite repression (Fig. 3E).

**FIG 4:**
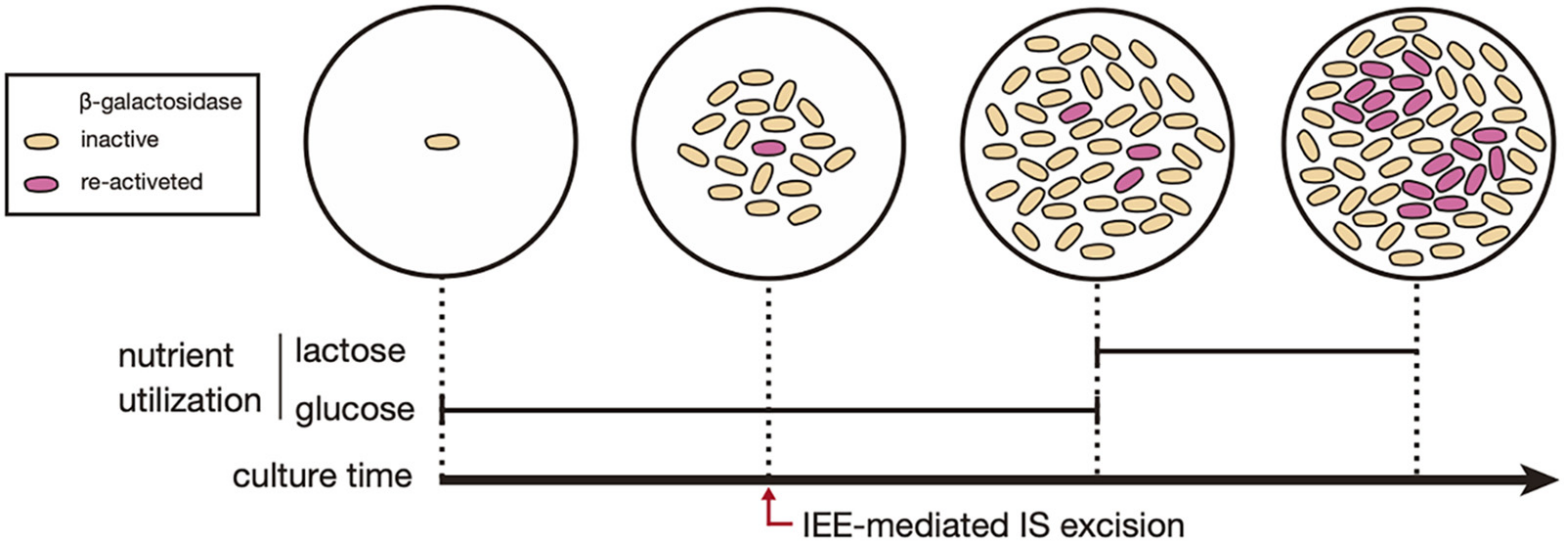
Schematic representation of the growth of E. coli harboring the *lacZ*^IS^ and *iee*^WT^ genes during cultivation in a medium supplemented with lactose and glucose.

In summary, we concluded that lactose does not affect IEE-mediated IS*600* excision, but the presence of lactose in culture medium promotes the selective proliferation of subclones carrying the IS*600*-excised *lacZ* gene, resulting in the formation of lactose-utilizing microcolonies after extended incubation, as illustrated in Fig. 4, which was recognized as the DLU phenotype.

### Variable IS insertion into the *lacZ* and *iee* genes in the O121:H19 lineage.

**FIG 5:**
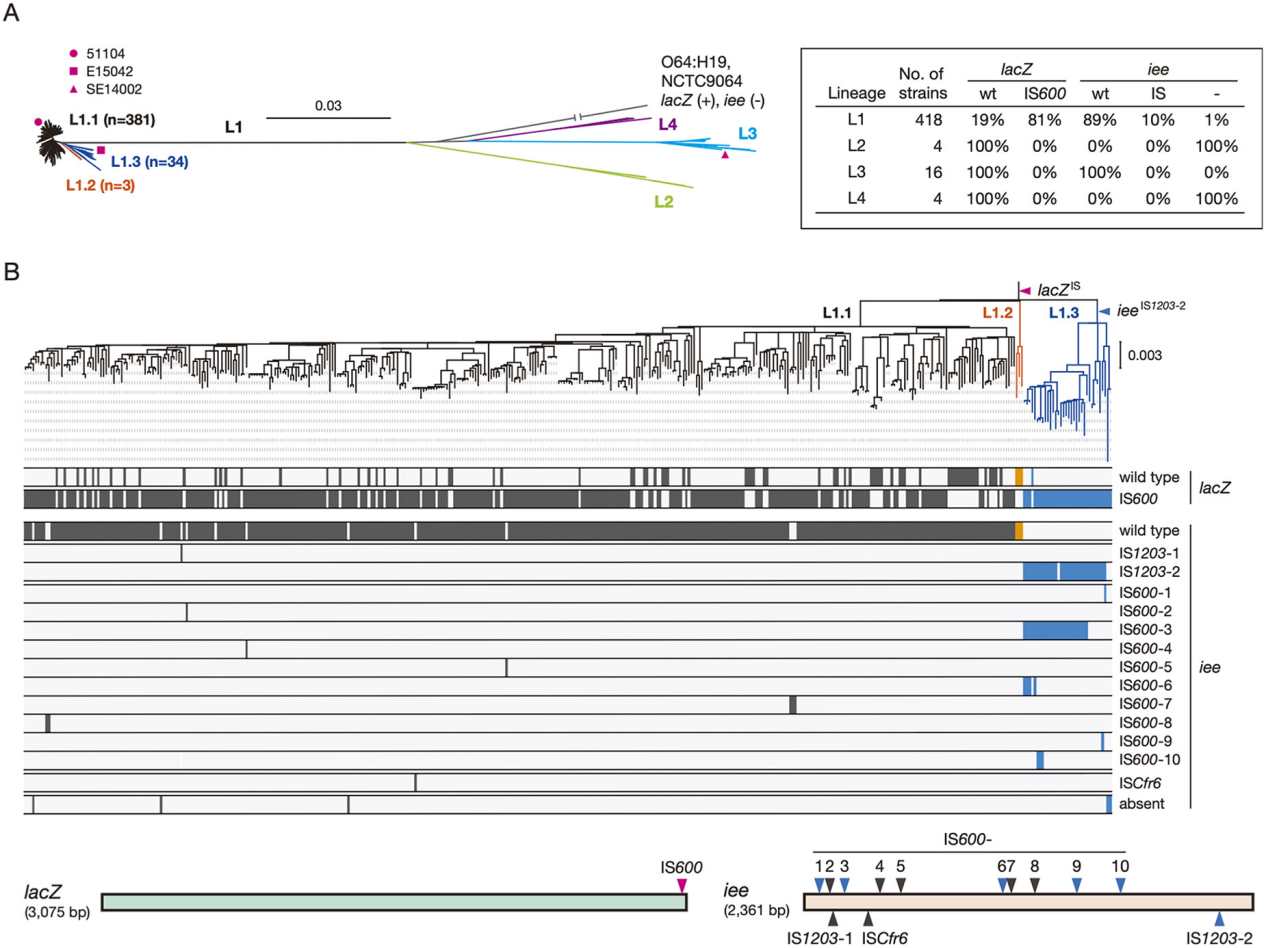
Phylogeny of STEC O121:H19 and variable IS insertion into the *lacZ* and *iee* genes. (A) Phylogenetic relationship of the 442 O121:H19 strains analyzed. This unrooted ML tree was taken from our previous paper (13) and modified. Three sublineages in the L1 lineage defined in this study (L1.1, L1.2, and L1.3) are indicated, and the numbers of strains belonging to each sublineage are shown in parentheses. The numbers of strains and the proportions of wild-type or IS-inserted *lacZ* and *iee* genes in the four lineages are presented in the inset. In the genome sequence of an L1.1 strain (FWSEC0155), the *iee* gene was split, but IS insertion was not detected; therefore, this strain was excluded from calculation. (B) IS insertion into the *lacZ* and *iee* genes in lineage L1. An ML tree of 418 L1 strains was constructed based on the recombination-free SNPs (7,591 sites) identified on the chromosomal backbone sequence (3,704,750 bp) using an L3 strain (SE14002) as the outgroup. IS elements inserted into *lacZ* and *iee* in each strain were mapped on the tree. As shown in the lower panel, where IS positions in *lacZ* and *iee* are indicated by colored triangles, the identified IS elements were distinguished based on their insertion position.

As our preliminary inspection of O121:H19 genomes suggested that there is some variation in IS insertion into the *lacZ* and *iee* genes among O121:H19 strains, the DLU phenotype appears to be a strain- or lineage-associated phenotype. To understand the phylogenetic background underlying the appearance of the DLU phenotype, we analyzed the variation in IS insertion into the *lacZ* and *iee* genes in the 442 O121:H19 strains (all were sequence type [ST] 655 or single locus variants of ST655) used in our previous phylogenetic analysis of the global O121:H19 population, in which O121:H19 strains were divided into four distinct lineages (L1-L4) (Data set S1) (13). This analysis revealed that IS insertion into the two genes occurred only in the major lineage, L1 (Fig. 5A). Although IS*600* insertion into *lacZ* was found in 81% of the L1 strains (338/418), IS insertion into *iee* (or the absence of *iee*) was detected in only 11% (48/418) of the L1 strains, and the remaining strains contained an intact *iee*. Therefore, most L1 strains exhibit the DLU phenotype upon extended cultivation in a lactose-supplemented medium, such as MAC.

We performed a more detailed analysis of the IS insertion into *lacZ* and *iee* in the L1 lineage. This lineage was divided into three sublineages (referred to as L1.1, L1.2, and L1.3) (Fig. 5), and of the three strains used for the above-mentioned analyses, strains 51104 and E15042 belonged to L1.1 and L1.3, respectively (strain SE14002 belonged to L3). While both *lacZ* and *iee* were intact in all L1.2 strains (*n* = 3), IS*600* insertion into *lacZ* was detected in most L1.1 and L1.3 strains (305/381 and 33/34, respectively) at the same position (Fig. 5B). This indicates that IS*600* insertion into *lacZ* occurred in the common ancestor of L1, and the IS*600* copy was deleted in sublineage L1.2 by the function of IEE. Although *iee* was present in all L1.1 and L1.3 strains except for five (3 in L1.1 and 2 in L1.3, in which the *iee*-encoding SpLE1-like integrative element appears to have been deleted), the pattern of IS insertion into *iee* was very different from that in *lacZ* (Fig. 5B). In L1.1, IS insertion into *iee* was detected sporadically (in only 10 strains), and the types and positions of IS varied between strains (black triangles in Fig. 5B), suggesting the independent insertion of these ISs in these strains. In contrast, the IS*1203* insertion found in strain E15042 (indicated as *iee*^IS^*^1203^*^-2^ in Fig. 5B) was detected in most *iee*-positive L1.3 strains (31/32) (Fig. 5B and Data set S1). Interestingly, one or two additional IS insertions into *iee* were found in many of the *iee*^IS^*^1203^*^-2^-carrying strains (26/32, at five insertion sites; all were IS*600* insertions), although the biological significance of this IS accumulation is currently unknown. IS*600* insertion was also found in an *iee*^IS^*^1203^*^-2^-negative L1.3 strain; thus, *iee* was inactivated by IS insertion in all L1.3 strains analyzed here. These results suggest that among the L1 strains, most L1.1 strains exhibit the DLU phenotype, but the L1.2 (*lacZ* is intact) and L1.3 strains (in which *iee* has been inactivated) do not exhibit this phenotype.

## DISCUSSION

This study revealed that the IEE-mediated excision of IS*600* from *lacZ* is responsible for the DLU phenotype observed in STEC O121:H19. Although IS*600* excision occurs at a low frequency, subclones carrying IS*600*-excised *lacZ* can selectively proliferate by using lactose during extended incubation, which explains why this phenotype can be observed only on lactose-containing agar plates. As the emergence of lactose-negative colonies from IS-cured subclones was not observed even after repeated subculturing on MAC agar plates, reversion to the negative phenotype is a very rare event at least *in vitro*. Although inactivation of the *lacZ* gene and switching lactose fermentation phenotype may confer some metabolic advantages to the O121:H19 strains carrying the IS*600*-inserted *lacZ* gene in some environments where carbon sources other than lactose are enriched, actual metabolic advantages by this mechanism is unknown.

O121:H19 STEC is one of the major STECs along with the O157, O26, O103, O111, O145, and O45 STECs (24) and is causing many outbreaks and sporadic cases of infection in the USA (25), Canada (26), and Japan (27). Of the four lineages of O121:H19, the major lineage (L1) is a globally circulating strain (13). The common ancestor of L1 strains acquired several virulence gene-encoding mobile genetic elements, such as the locus of enterocyte effacement (LEE), a virulence plasmid, and the Stx2 phage (13). Our current analysis revealed that IS*600* insertion into *lacZ* also occurred in the common ancestor of L1 and that the *iee* gene is intact in most strains belonging to the L1.1 lineage, which is the major sublineage of L1 (Fig. 5B). Thus, the majority of O121:H19 strains isolated worldwide exhibit the DLU phenotype, although we should remember that the L2, L3, and L4 strains and the L1.2 strains show typical lactose fermentation and that the L1.3 strains and a few L1.1 strains are negative for lactose fermentation. These data are helpful in the screening, isolation, and identification of O121:H19 STEC.

We found that the cultivation of bacteria carrying both *iee* and IS*3* family members easily generates phenotypic changes in the population. In the O121:H19 strain 51104, a total of 27 copies of IS*600* (22 on the chromosome and five on the plasmid) as well as multiple intact copies of other IS*3* family members, such as IS*629* (10 copies) and IS*Cfr6* (six copies) were present (13). Thus, IEE-mediated phenotype changes other than that of lactose fermentation could take place in strain 51104 and probably in other O121:H19 strains containing the intact *iee* gene. Major STECs contain *iee* and multiple copies of IS*3* family members, such as IS*629* and IS*600* (which are often extensively amplified), as does ETEC O139 isolated from swine (12–15, 28). Therefore, gene inactivation/reactivation by IS elements and IEE can occur also in these STEC and ETEC strains, which may cause changes in various phenotypes in nature and during cultivation in the laboratory. Moreover, as many IEE homologs have been identified in a broad range of bacterial species (11), although their functions have yet to be analyzed, IEE-mediated IS excision may also play an important role in phenotypic changes in other bacterial species.

## MATERIALS AND METHODS

### Bacterial strains and media.

The E. coli strains and their mutants used in this study are listed in Table 1. Whole-genome sequences of the 442 O121:H19 strains (Data set S1) previously used in our phylogenetic analysis (13) were used for the analysis of IS insertion into the *lacZ* and *iee* genes.

Bacteria were grown in the following media: LB (1% [wt/vol] Bacto Tryptone, Gibco; 0.5% [wt/vol] Bacto Yeast Extract, Becton, Dickinson [BD]; 1% [wt/vol] sodium chloride, nacalai tesque), LB agar (LB containing 1.5% [wt/vol] Bacto Agar, BD), MAC (Difco MacConkey agar base, BD; 1% [wt/vol] lactose monohydrate, Wako), MAC not supplemented with lactose (Difco MacConkey agar base), Pearlcore MAC (Pearlcore MacConkey agar, Eiken Chemical Co.), MM (Difco M9 Minimal Salts, BD; 2 mM magnesium sulfate heptahydrate, Wako; 0.1% D-[+]-glucose, nacalai tesque), and MM agar (MM containing 1.5% [wt/vol] Bacto Agar). The growth media were supplemented with regents and antibiotics when necessary at the following concentrations: L(+)-arabinose (Wako), 1 mM; IPTG (Wako), 0.3 mM or 30 mM; X-gal (TaKaRa), 40 μg/mL; sucrose (nacalai tesque), 10% (wt/vol); D-(+)-glucose, 0.1%, 0.2%, or 0.4% (wt/vol); lactose monohydrate, 1.0% (wt/vol); D(+)-maltose monohydrate (Wako), 1.0% (wt/vol); chloramphenicol (Wako), 20 μg/mL; ampicillin (Sigma), 50 μg/mL; tetracycline (nacalai tesque), 10 μg/mL.

### Construction of mutant strain.

Mutant strains were generated as follows using the plasmid vectors and primers listed in Tables 2 and 3, respectively. To disrupt the *iee* gene in the O121:H19 strain 51104, we first introduced the Red recombinase-encoding pKD46 (29) into the strain. A DNA fragment containing the chloramphenicol resistance (Cm^R^) cassette and terminal 55-nt extensions homologous to the *iee*-flanking region was generated by the 2-step tailed-PCR method using the two sets of primers (iee-H1-F1/R1 and iee-H1-F2/R2) and pKD3 as a template. The 1.1-kbp PCR product was purified, treated with DpnI, and transformed into 51104 carrying pKD46, in which arabinose-inducible Red recombinase was expressed. Disruption of the *iee* gene in Cm^R^ transformants was confirmed by colony PCR using EmeraldAmp MAX PCR Master Mix (TaKaRa) and specific primers (Viee-F/R).

**TABLE 2:** Primers used to generate mutant strains

Name	Sequence (5′–3′)	Regions/positions of boldfaced sequences
*iee* gene disruption primers		
iee-H1-F1	AACCCGGGGAGCTCAAATTATTTA- AAAA**GTGTAGGCTGGAGCTGCTTC**	pKD3
iee-H1-R1	TCAGTGGATCGGATACAGGTAAC- GAAT**ATGGGAATTAGCCATGGTCC**	pKD3
iee-H1-F2	TATACTGGGACTCTTTGTTGCCC- GAAC**AACCCGGGGAGCTCAAAT**	5′-terminal region of the PCR product amplified with the iee-H1-F1 and -R1 primers
iee-H1-R2	AGGGGACCCGAATTTCTGCCGTG- GTGCCT**CAGTGGATCGGATACA**	3′-terminal region of the PCR product amplified with the iee-H1-F1 and -R1 primers
Primers to verify disruptions		
Viee-F	GTTCTTACTGCCGGTAGCCATT	
Viee-R	CCTCATTACAGTAATGCGGT	
Primers to construct the lacZ^IS^-pABB-CRS2 plasmid		
lacZ^IS^-UF	**AGAGCTCGGATCCACTAG**CGTAG- TGCAACCGAACGCGA	Terminus of the XbaI and SpeI-digested pABB-CRS2 plasmid
lacZ^IS^-UR	**GGTAATGGTAGCGACC**GGCGCTCA	5′-terminal region of the PCR product amplified with the lacZ^IS^-F and -R primers
lacZ^IS^-F	**GGTCGCTACCATTACC**TGAG	3′-terminal region of the PCR product amplified with the lacZ^IS^-UF and -UR primers
lacZ^IS^-R	**CACCAGACCAACTGGT**TGAG	5′-terminal region of the PCR product amplified with the lacZ^IS^-DF and -DR primers
lacZ^IS^-DF	**AAAGGATCGATCCTCTAG**ACAG- ACATAATAGTGCCAGC	3′-terminal region of the PCR product amplified with the lacZ^IS^-F and -R primers
lacZ^IS^-DR	**ACCAGTTGGTCTGGTG**TCAA	Terminus of the XbaI and SpeI-digested pABB-CRS2 plasmid
Primers to verify the insertion of three amplicons into the *Xba*I and *Spe*I-digested pABB-CRS2 plasmid		
VlacZ^IS^-pABB-CRS2-F	GTTGCATGGGCATAAAGTTG	
VlacZ^IS^-pABB-CRS2-R	CGGCTGACATGGGAATTCTA	
Primers to verify integration of the lacZ^IS^-pABB-CRS2 plasmid		
Vint-lacZ^IS^-pABB-CRS2-F1	GGGAAGTAGGCTCCCATGA	
Vint-lacZ^IS^-pABB-CRS2-R1	CCTCTGGATGTCGCTCCACA	
Vint-lacZ^IS^-pABB-CRS2-F2	CAACTGATGGAAACCAGCC	
Vint-lacZ^IS^-pABB-CRS2-R2	CTGAGGTGGCGAACGATGAG	
Primers to verify the insertion of IS*600* into the *lacZ*		
VlacZ^IS^-F	GGGAAGTAGGCTCCCATGA	
VlacZ^IS^-R	CAACTGATGGAAACCAGCC	

**TABLE 3:** Plasmids used to generate mutant strains

Plasmids	Description^*a*^	Source or reference
pKD46	Ap^R^; oriR101-derived Red recombinase expression plasmid	29
pKD3	Cm^R^; cloning vector	29
pABB-CRS2	Ap^R^, Cm^R^; R6K-derived suicide vector	30
lacZ^IS^-pABB-CRS2	Ap^R^; 51104 IS*600*-inserted *lacZ* cloned into pABB-CRS2	This study

a Ap^R^, ampicillin resistance; Cm^R^, chloramphenicol resistance.

To generate a K-12 mutant that carried the same *lacZ*^IS^ gene as strain 51104 (K-12_*lacZ*^IS^), we constructed a pABB-CRS2-based suicide vector (30), as shown in Fig. S4. Flanking regions of IS*600* in the *lacZ* were amplified using two sets of primers (lacZ^IS^-UF/UR and lacZ^IS^-DF/DR) that included a 3-nt sequence of the target sequence for IS*600* or an 18-nt sequence homologous to pABB-CRS2 at each 5′ end and the genomic DNA of K-12 as a template to obtain two amplicons. The IS*600* sequence with a 13-bp sequence homologous to these two amplicons at each terminal end was also amplified using primers lacZ^IS^-F/R and the genomic DNA of strain 51104 as a template. These three amplicons were purified by gel extraction and cloned into XbaI- and SpeI-digested pABB-CRS2 by the Red/ET recombination-based seamless DNA cloning method (SLiCE method) (31). The recombinant product was introduced into One Shot PIR1 Chemically Competent E. coli (Invitrogen) by transformation. Clones were selected on LB agar containing ampicillin (Ap plate). The insertion of the amplicons into pABB-CRS2 was confirmed by colony PCR using specific primers (VlacZ^IS^-pABB-CRS2-F/R). The purified plasmid (lacZ^IS^-pABB-CRS2) was transformed into K-12 using an electroporator (Gene Pulser II; Bio–Rad), followed by selection on Ap plates. Chromosome integration of lacZ^IS^-pABB-CRS2 by a single crossover was confirmed by PCR using two sets of primers (Vint-lacZ^IS^-pABB-CRS2-F1/R1 and Vint-lacZ^IS^-pABB-CRS2-F2/R2), and total cellular DNA was extracted from isolated colonies using the alkaline-boiling method. To allow the excision of the *sacB*-containing vector, the transformants were grown in LB containing sucrose without NaCl for 6 h at 30°C with shaking and spread on LB agar plates containing sucrose without NaCl at 30°C. Finally, the presence of the *lacZ*^IS^ gene in each sucrose-resistant and Ap-sensitive colony was confirmed by PCR using specific primers (VlacZ^IS^-F/R).

We constructed pBR-*iee* using pBR322 (Accession No. J01749; TaKaRa) by a similar strategy to that described for lacZ^IS^-pABB-CRS2 using the primers listed in Table 4. The *iee*-encoding region was amplified using the genomic DNA of the O157:H7 strain Sakai (32) as a template and primers that included 15-nt sequences homologous to pBR322 at each 5′ end (ECs1305-pBR-F/R). The amplicon was purified by gel extraction and cloned into EcoRI/PstI-digested pBR322 by the SLiCE method. The recombinant product was introduced into NEB 5α (New England Biolabs) by transformation. Clones were selected on LB agar containing tetracycline. Insertion of the amplicon into pBR322 was confirmed by colony PCR using specific primers (VpBR-iee-F/R). pBR322 and pBR-*iee* were introduced into K-12_*lacZ*^IS^ using an electroporator (Gene Pulser II), and plasmid-carrying clones were selected based on tetracycline resistance.

**TABLE 4:** Primers used to construct pBR-*iee*^*a*^

Name	Sequence (5′–3′)
Primers to construct pBR-*iee* plasmid	
ECs1305-pBR-F	**TCAAACATGAGAATT**CTGAATTACAGCTGTATTAGG
ECs1305-pBR-R	**GTTGCCATTGCTGCA**TTTTTGATGACGTTCTCACTG
Primers to verify the insertion of *iee* and its flanking region into pBR322 plasmid	
VpBR-iee-F	CGTGTTATAGGTTGTGCGGAGA
VpBR-iee-R	TTTGCAAGCAGCAGATTACG

a The boldfaced sequence in ECs1305-pBR-F and -R correspond to each terminal of the EcoRI and PstI-digested pBR322 plasmid.

### Analysis of lactose fermentation, bacterial growth, and the copy number of *lacZ* gene.

Seven experiments outlined in Fig. S5 were performed in this study. In all experiments, tetracycline (10 μg/mL at the final concentration) was added to the culture medium for the maintenance of pBR322 and pBR-*iee* in K-12_*lacZ*^IS^.

**Experiment 1**. Three O121:H19 strains (51104, E15042, and SE14002) were streaked onto a MacConkey agar base supplemented with lactose (MAC plates) and cultured for 40 h at 37°C. Then, single colonies were subcultured on MAC for 16 h at 37°C. Red or white colonies (lactose fermenting or not, respectively) were suspended in LB, and total cellular DNA was extracted from the suspension using the alkaline-boiling method and used for PCR analysis with the primers shown in Fig. S5. The remaining suspension was diluted to 1 × 10^9^ CFU/mL (1 OD_600_), and the diluent (5 μL) was spotted onto LB agar supplemented with 0.3 mM IPTG (Wako) and X-gal (TaKaRa) and cultured for 16 h at 37°C. The β-galactosidase activity in each suspension was judged by the hydrolysis of X-gal (blue colored spot). Two *lacY*-deficient K-12 derivatives carrying the wild-type or degraded *lacZ* gene (HB101 or JM109, respectively; TaKaRa) were used as controls.

**Experiment 2.** Four strains (O121:H19 51104, 51104Δ*iee*, K-12_*lacZ*^IS^:pBR, and K-12_*lacZ^I^*^S^:p*iee*) were streaked onto MAC and MacConkey agar base plates and cultured for 40 h at 37°C. Five colonies on each plate were randomly selected and suspended in LB. Clones in these suspensions were examined by the following procedures:

(i) The suspensions were diluted to 1~2 × 10^3^ CFU/mL, and each diluent (100 μL) was inoculated onto Pearlcore MAC plates. After incubation for 16 h at 37°C, the numbers of red and white colonies were counted.
(ii) The suspensions were diluted to 2.5 × 10^9^ CFU/mL, and each diluent (5 μL) was spotted onto Pearlcore MAC plates and cultured for 16 h at 37°C.

**Experiment 3.** K-12_*lacZ*^IS^:p*iee* cultured for 8 h in LB at 37°C were inoculated in 2 mL of LB or LB supplemented with 1% (equivalent to 30 mM) lactose, 30 mM IPTG or 0.3 mM IPTG at 0.1 OD_600_ and grown for 18 h at 37°C with shaking. Clones in the cultures were examined by the same procedures as in Experiments 2-i and 2-ii.

**Experiment 4.** K-12_*lacZ*^IS^:p*iee* cultured for 8 h in LB at 37°C were inoculated in 5 mL of LB or LB supplemented with lactose or maltose at 0.1 OD_600_ and grown for 24 h at 37°C with shaking. At each time point, the OD_600_ of each culture was determined, and bacterial cells were collected to purify total cellular DNA using the DNeasy blood and tissue kit (Qiagen). Using cellular DNA, the copy number of IS*600*-excised *lacZ* was determined by droplet digital PCR using the EvaGreen assay (Bio–Rad) with *lacZ*-specific primers (5′-AGCCGCTACAGTCAACAGCA-3′ and 5′-ACGCGAAATACGGGCAGACA-3′). The copy number relative to that of *lacY* was determined by dividing the copy number of IS*600*-excised *lacZ* by that of *lacY*. The *lacY* gene was amplified with the following specific primers: 5′-AGTAAAACGGCGAGGATGAGTG-3′ and 5′-GCGGATGTTTGGCTGTGTTG-3′.

**Experiment 5.** K-12_*lacZ*^IS^:p*iee* cultured for 8 h in LB at 37°C were inoculated into 2 mL of LB supplemented with lactose and several concentrations (0%, 0.1%, 0.2%, and 0.4%) of glucose and grown for 18 h at 37°C with shaking. Clones in the cultures were examined by counting red or white colonies as described for Experiment 2-i.

**Experiment 6.** K-12_*lacZ*^IS^:p*iee* was streaked onto MM agar and cultured for 16 h at 37°C. Colonies suspended in MM were inoculated in 5 mL of MM or MM supplemented with lactose or maltose at 0.05 OD_600_ and grown for 48 h at 37°C with shaking. At each time point, the OD_600_ of each culture and the copy number of IS*600*-excised *lacZ* and *lacY* in bacterial cells were determined as described for Experiment 4.

**Experiment 7.** K-12_*lacZ*^IS^:p*iee* was streaked onto MM agar and cultured for 16 h at 37°C. Colonies suspended in MM were inoculated in 2 mL of MM supplemented with lactose and several concentrations (0%, 0.1%, and 0.2%) of glucose at 0.05 OD_600_ and grown for 36 h at 37°C with shaking. Clones in the cultures were examined by counting red or white colonies as described for Experiment 2-i.

### Analysis of the IS insertion into *lacZ* and *iee* among O121:H19 genomes.

Strategies for searching the IS insertion into *lacZ* and *iee* are outlined in Fig. S6. Briefly, the *lacZ* (locus tag: SE14002_0329 in AP024473) and *iee* (SE14002_1200 in AP024473 and EC51104_3767 in AP024471) genes in 433 O121:H19 draft genomes were first identified by a BLATN-based search. When a gene split by some insertion was detected, we examined the presence and type of inserted IS using the ISCompare program (33) with the ISFinder database (access on Oct. 2020; https://github.com/thanhleviet/ISfinder-sequences) (2).

### SNP detection and phylogenetic analysis.

SNP sites (7,591 sites) of the core genome sequences of 419 O121:H19 strains (418 L1 strains and one L3 strain used as the outgroup) were detected by MUMmer (34). After removing recombinogenic SNPs by Gubbins (35), they were used to construct an ML tree in RAxML (36) with the GTR gamma substitution model as previously described (13). ML trees were displayed and annotated using iTOL (37).

## References

[1] Siguier P, Gourbeyre E, Varani A, Ton-Hoang B, Chandler M. 2015. Everyman’s guide to bacterial insertion sequences. Microbiol Spectr 3:MDNA3. MDNA3-0030–2014. 10.1128/microbiolspec.MDNA3-0030-2014. 26104715

[2] Siguier P, Perochon J, Lestrade L, Mahillon J, Chandler M. 2006. ISfinder: the reference centre for bacterial insertion sequences. Nucleic Acids Res 34:D32–36. 10.1093/nar/gkj014. 16381877 PMC1347377

[3] Wei J, Goldberg MB, Burland V, Venkatesan MM, Deng W, Fournier G, Mayhew GF, Plunkett G, 3rd, Rose DJ, Darling A, Mau B, Perna NT, Payne SM, Runyen-Janecky LJ, Zhou S, Schwartz DC, Blattner FR. 2003. Complete genome sequence and comparative genomics of *Shigella flexneri* serotype 2a strain 2457T. Infect Immun 71:2775–2786. 10.1128/IAI.71.5.2775-2786.2003. 12704152 PMC153260

[4] Kothapalli S, Nair S, Alokam S, Pang T, Khakhria R, Woodward D, Johnson W, Stocker BAD, Sanderson KE, Liu S-L. 2005. Diversity of genome structure in *Salmonella enterica* serovar Typhi populations. J Bacteriol 187:2638–2650. 10.1128/JB.187.8.2638-2650.2005. 15805510 PMC1070368

[5] Ooka T, Ogura Y, Asadulghani M, Ohnishi M, Nakayama K, Terajima J, Watanabe H, Hayashi T. 2009. Inference of the impact of insertion sequence (IS) elements on bacterial genome diversification through analysis of small-size structural polymorphisms in *Escherichia coli* O157 genomes. Genome Res 19:1809–1816. 10.1101/gr.089615.108. 19564451 PMC2765283

[6] Hawkey J, Monk JM, Billman-Jacobe H, Palsson B, Holt KE. 2020. Impact of insertion sequences on convergent evolution of *Shigella* species. PLoS Genet 16:e1008931. 10.1371/journal.pgen.1008931. 32644999 PMC7373316

[7] Vandecraen J, Chandler M, Aertsen A, Van Houdt R. 2017. The impact of insertion sequences on bacterial genome plasticity and adaptability. Crit Rev Microbiol 43:709–730. 10.1080/1040841X.2017.1303661. 28407717

[8] Amman F, D'Halluin A, Antoine R, Huot L, Bibova I, Keidel K, Slupek S, Bouquet P, Coutte L, Caboche S, Locht C, Vecerek B, Hot D. 2018. Primary transcriptome analysis reveals importance of IS elements for the shaping of the transcriptional landscape of *Bordetella pertussis*. RNA Biol 15:967–975. 10.1080/15476286.2018.1462655. 29683387 PMC6161684

[9] Kusumoto M, Nishiya Y, Kawamura Y. 2000. Reactivation of insertionally inactivated Shiga toxin 2 genes of *Escherichia coli* O157:H7 caused by nonreplicative transposition of the insertion sequence. Appl Environ Microbiol 66:1133–1138. 10.1128/AEM.66.3.1133-1138.2000. 10698782 PMC91953

[10] Whiteway C, Valcek A, Philippe C, Strazisar M, De Pooter T, Mateus I, Breine A, Van der Henst C. 2022. Scarless excision of an insertion sequence restores capsule production and virulence in Acinetobacter baumannii. ISME J 16:1473–1477. 10.1038/s41396-021-01179-3. 34949784 PMC9038732

[11] Kusumoto M, Ooka T, Nishiya Y, Ogura Y, Saito T, Sekine Y, Iwata T, Akiba M, Hayashi T. 2011. Insertion sequence-excision enhancer removes transposable elements from bacterial genomes and induces various genomic deletions. Nat Commun 2:152. 10.1038/ncomms1152. 21224843

[12] Nakamura K, Murase K, Sato MP, Toyoda A, Itoh T, Mainil JG, Piérard D, Yoshino S, Kimata K, Isobe J, Seto K, Etoh Y, Narimatsu H, Saito S, Yatsuyanagi J, Lee K, Iyoda S, Ohnishi M, Ooka T, Gotoh Y, Ogura Y, Hayashi T. 2020. Differential dynamics and impacts of prophages and plasmids on the pangenome and virulence factor repertoires of Shiga toxin-producing *Escherichia coli* O145:H28. Microb Genom 6:e000323. 10.1099/mgen.0.000323. PMC7067040 31935184

[13] Nishida R, Nakamura K, Taniguchi I, Murase K, Ooka T, Ogura Y, Gotoh Y, Itoh T, Toyoda A, Mainil JG, Piérard D, Seto K, Harada T, Isobe J, Kimata K, Etoh Y, Hamasaki M, Narimatsu H, Yatsuyanagi J, Kameyama M, Matsumoto Y, Nagai Y, Kawase J, Yokoyama E, Ishikawa K, Shiomoto T, Lee K, Kang D, Akashi K, Ohnishi M, Iyoda S, Hayashi T. 2021. The global population structure and evolutionary history of the acquisition of major virulence factor-encoding genetic elements in Shiga toxin-producing *Escherichia coli* O121:H19. Microb Genom 7:e000716. 10.1099/mgen.0.000716 PMC8767318 34878971

[14] Ogura Y, Ooka T, Iguchi A, Toh H, Asadulghani M, Oshima K, Kodama T, Abe H, Nakayama K, Kurokawa K, Tobe T, Hattori M, Hayashi T. 2009. Comparative genomics reveal the mechanism of the parallel evolution of O157 and non-O157 enterohemorrhagic *Escherichia coli*. Proc Natl Acad Sci USA 106:17939–17944. 10.1073/pnas.0903585106. 19815525 PMC2764950

[15] Kusumoto M, Fukamizu D, Ogura Y, Yoshida E, Yamamoto F, Iwata T, Ooka T, Akiba M, Hayashi T. 2014. Lineage-specific distribution of insertion sequence excision enhancer in enterotoxigenic *Escherichia coli* isolated from swine. Appl Environ Microbiol 80:1394–1402. 10.1128/AEM.03696-13. 24334665 PMC3911043

[16] Lorenz SC, Gonzalez-Escalona N, Kotewicz ML, Fischer M, Kase JA. 2017. Genome sequencing and comparative genomics of enterohemorrhagic *Escherichia coli* O145:H25 and O145:H28 reveal distinct evolutionary paths and marked variations in traits associated with virulence & colonization. BMC Microbiol 17:183. 10.1186/s12866-017-1094-3. 28830351 PMC5567499

[17] Ooka T, Terajima J, Kusumoto M, Iguchi A, Kurokawa K, Ogura Y, Asadulghani M, Nakayama K, Murase K, Ohnishi M, Iyoda S, Watanabe H, Hayashi T. 2009. Development of a multiplex PCR-based rapid typing method for enterohemorrhagic *Escherichia coli* O157 strains. J Clin Microbiol 47:2888–2894. 10.1128/JCM.00792-09. 19641072 PMC2738077

[18] Stanton E, Park D, Döpfer D, Ivanek R, Kaspar CW. 2014. Phylogenetic characterization of *Escherichia coli* O157:H7 based on IS*629* distribution and Shiga toxin genotype. Microbiology (Reading) 160:502–513. 10.1099/mic.0.073437-0. 24425770

[19] Batt CA, Tortorello ML. 2014. Encyclopedia of food microbiology 2nd ed Academic Press, San Diego CA.

[20] Gottschalk G. 1985. Bacterial metabolism, 2nd ed Springer-Verlag, New York.

[21] Gill A, McMahon T, Dussault F, Jinneman K, Lindsey R, Martin H, Stoneburg D, Strockbine N, Wetherington J, Feng P. 2022. Delayed lactose utilization among Shiga toxin-producing *Escherichia coli* of serogroup O121. Food Microbiol 102:103903. 10.1016/j.fm.2021.103903. 34809935

[22] Boos W, Shuman H. 1998. Maltose/maltodextrin system of *Escherichia coli*: transport, metabolism, and regulation. Microbiol Mol Biol Rev 62:204–229. 10.1128/MMBR.62.1.204-229.1998. 9529892 PMC98911

[23] Görke B, Stülke J. 2008. Carbon catabolite repression in bacteria: many ways to make the most out of nutrients. Nat Rev Microbiol 6:613–624. 10.1038/nrmicro1932. 18628769

[24] Brooks JT, Sowers EG, Wells JG, Greene KD, Griffin PM, Hoekstra RM, Strockbine NA. 2005. Non-O157 Shiga toxin-producing *Escherichia coli* infections in the United States, 1983–2002. J Infect Dis 192:1422–1429. 10.1086/466536. 16170761

[25] Crowe SJ, Bottichio L, Shade LN, Whitney BM, Corral N, Melius B, Arends KD, Donovan D, Stone J, Allen K, Rosner J, Beal J, Whitlock L, Blackstock A, Wetherington J, Newberry LA, Schroeder MN, Wagner D, Trees E, Viazis S, Wise ME, Neil KP. 2017. Shiga toxin–producing *E. coli* infections associated with flour. N Engl J Med 377:2036–2043. 10.1056/NEJMoa1615910. 29166238 PMC5792826

[26] Morton V, Cheng JM, Sharma D, Kearney A. 2017. Outbreak detection: an outbreak of Shiga toxin-producing *Escherichia coli* O121 infections associated with flour—Canada, 2016–2017. Can Commun Dis Rep 43:154–155. 10.14745/ccdr.v43i78a03. 29770083 PMC5864315

[27] Kikuchi K, Lee K, Ueno H, Tomari K, Kobori S, Kaetsu A, Matsui M, Suzuki S, Sekizuka T, Kuroda M, Miyazaki M, Ohnishi M. 2019. Enterohaemorrhagic *Escherichia coli* O121:H19 acquired an extended-spectrum β-lactamase gene during the development of an outbreak in two nurseries. Microb Genom 5:569. 10.1099/mgen.0.000278. PMC6700663 31215859

[28] Toro M, Rump LV, Cao G, Meng J, Brown EW, Gonzalez-Escalona N. 2015. Simultaneous presence of insertion sequence excision enhancer and insertion sequence IS*629* correlates with increased diversity and virulence in Shiga toxin-producing *Escherichia coli*. J Clin Microbiol 53:3466–3473. 10.1128/JCM.01349-15. 26292302 PMC4609730

[29] Datsenko KA, Wanner BL. 2000. One-step inactivation of chromosomal genes in *Escherichia coli* K-12 using PCR products. Proc Natl Acad Sci USA 97:6640–6645. 10.1073/pnas.120163297. 10829079 PMC18686

[30] Sekiya K, Ohishi M, Ogino T, Tamano K, Sasakawa C, Abe A. 2001. Supermolecular structure of the enteropathogenic *Escherichia coli* type III secretion system and its direct interaction with the EspA-sheath-like structure. Proc Natl Acad Sci USA 98:11638–11643. 10.1073/pnas.191378598. 11562461 PMC58782

[31] Motohashi K. 2015. A simple and efficient seamless DNA cloning method using SLiCE from *Escherichia coli* laboratory strains and its application to SLiP site-directed mutagenesis. BMC Biotechnol 15:47. 10.1186/s12896-015-0162-8. 26037246 PMC4453199

[32] Hayashi T, Makino K, Ohnishi M, Kurokawa K, Ishii K, Yokoyama K, Han C-G, Ohtsubo E, Nakayama K, Murata T, Tanaka M, Tobe T, Iida T, Takami H, Honda T, Sasakawa C, Ogasawara N, Yasunaga T, Kuhara S, Shiba T, Hattori M, Shinagawa H. 2001. Complete genome sequence of enterohemorrhagic *Eschelichia coli* O157:H7 and genomic comparison with a laboratory strain K-12. DNA Res 8:11–22. 10.1093/dnares/8.1.11. 11258796

[33] Mogro EG, Ambrosis NM, Lozano MJ. 2021. Easy identification of insertion sequence mobilization events in related bacterial strains with ISCompare. G3 11:jkab181. 10.1093/g3journal/jkab181. 34849821 PMC8496243

[34] Kurtz S, Phillippy A, Delcher AL, Smoot M, Shumway M, Antonescu C, Salzberg SL. 2004. Versatile and open software for comparing large genomes. Genome Biol 5:R12. 10.1186/gb-2004-5-2-r12. 14759262 PMC395750

[35] Croucher NJ, Page AJ, Connor TR, Delaney AJ, Keane JA, Bentley SD, Parkhill J, Harris SR. 2015. Rapid phylogenetic analysis of large samples of recombinant bacterial whole genome sequences using Gubbins. Nucleic Acids Res 43:e15. 10.1093/nar/gku1196. 25414349 PMC4330336

[36] Stamatakis A. 2006. RAxML-VI-HPC: maximum likelihood-based phylogenetic analyses with thousands of taxa and mixed models. Bioinformatics 22:2688–2690. 10.1093/bioinformatics/btl446. 16928733

[37] Letunic I, Bork P. 2019. Interactive Tree of Life (iTOL) v4: recent updates and new developments. Nucleic Acids Res 47:W256–W259. 10.1093/nar/gkz239. 30931475 PMC6602468

[38] Blattner FR, Plunkett G, 3rd, Bloch CA, Perna NT, Burland V, Riley M, Collado-Vides J, Glasner JD, Rode CK, Mayhew GF, Gregor J, Davis NW, Kirkpatrick HA, Goeden MA, Rose DJ, Mau B, Shao Y. 1997. The complete genome sequence of *Escherichia coli* K-12. Science 277:1453–1462. 10.1126/science.277.5331.1453. 9278503

