# Supplementary material for: Insertion Sequence (IS)-Excision Enhancer (IEE)-Mediated IS Excision from the lacZ Gene Restores the Lactose Utilization Defect of Shiga Toxin-Producing Escherichia coli O121:H19 Strains and Is Responsible for Their Delayed Lactose Utilization Phenotype
Source: Appl Environ Microbiol. 2022 Aug 1;88(16):e00760-22. doi: 10.1128/aem.00760-22 (PMC9397093; doi:10.1128/aem.00760-22)
Supplement: Supplemental file 1 — Fig. S1 to S6. Download aem.00760-22-s0001.pdf, PDF file, 1.7 MB [file aem.00760-22-s0001.pdf]

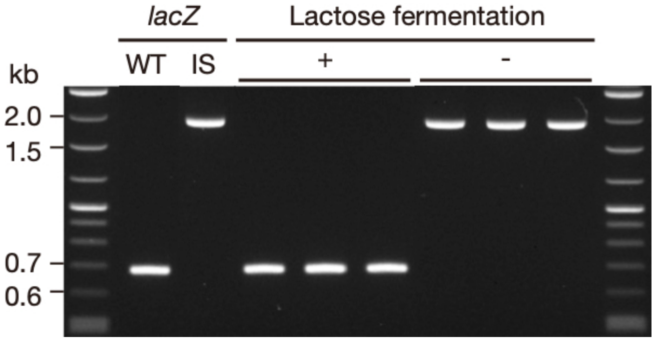

FIG S1. Detection of the IS*600*-excised *lacZ* gene in the subcultured colonies of O121:H19 strain 51104. The results of PCR analysis of total cellular DNA extracted from the colonies isolated from a single colony of strain 51104 cultured for 40 h on MAC plates are shown. See Fig. 1 for the details of each strain and colony. Total cellular DNA samples extracted from single colonies of strains SE14002 and 51104 ($lacZ^{WT}$ and $lacZ^{IS}$, respectively) cultured on LB plates were used as controls.

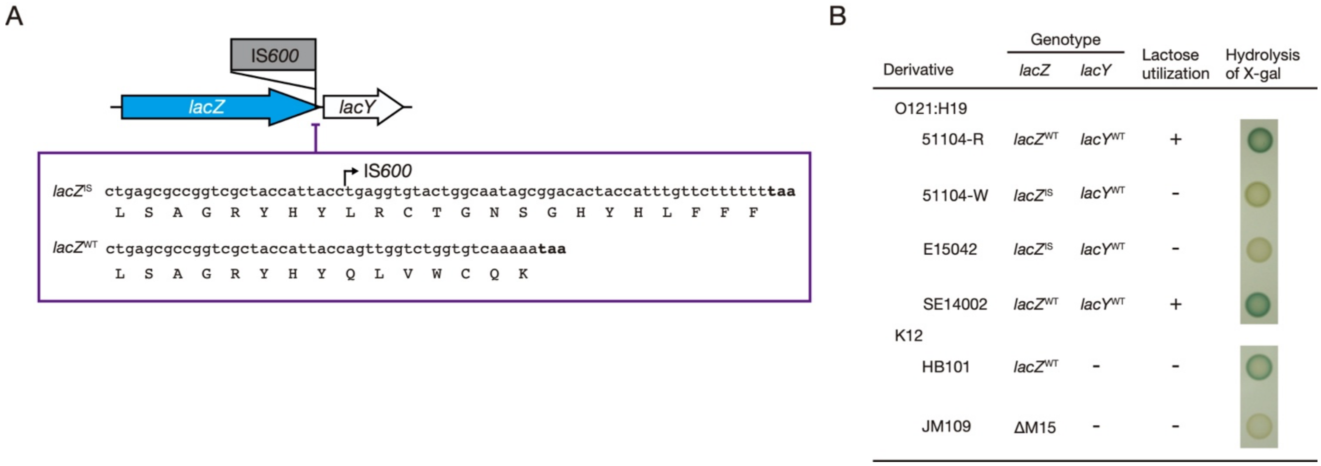

FIG S2. Examination of β-galactosidase activity in the *lacZ*^{IS}-harboring clone. (A) The position of IS*600* insertion into the *lacZ* gene. The 3'-end of the coding sequences and deduced amino acid sequences are shown. The stop codons are indicated by bold text. (B) Hydrolysis of 5-bromo-4-chloro-3-indolyl-β-D-galactopyranoside (X-gal) in O121:H19 and K-12 derivatives. See Fig. 1 for the details of each derivative. Lactose utilization was gauged by the colony color on MacConkey agar. The *lacZ* gene was induced by isopropyl-β-d-thiogalactopyranoside (IPTG) during cultivation for 16 h, and β-galactosidase activity was detected by the white-to-blue color changes of colonies grown on LB agar supplemented with 40 μg/ml of X-gal.

	Supplement for LB			
			IPTG	
	–	Lactose	30 mM	0.3 mM
No. of red colonies[a] (No. of total colonies)	1 (369)	181 (482)	0 (461)	1 (465)
Spot of bacterial cell suspension	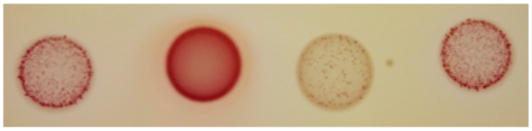			

[a] Total numbers of colonies formed on three independent experiments

FIG S3. The proportion of lactose-fermenting clones in the K-12_*lacZ*^{IS}:p*iee* culture grown in LB supplemented with IPTG. In the upper row, the number of colonies isolated from K-12_*lacZ*^{IS}:p*iee* cultured in the indicated medium for 18 h at 37 °C is shown. The lower row shows spots of each culture (approximately 1.3 x 10^7 CFU) grown on MAC plates for 16 h at 37 °C (representative images are presented here). Cultures grown in LB with or without lactose were used as controls.

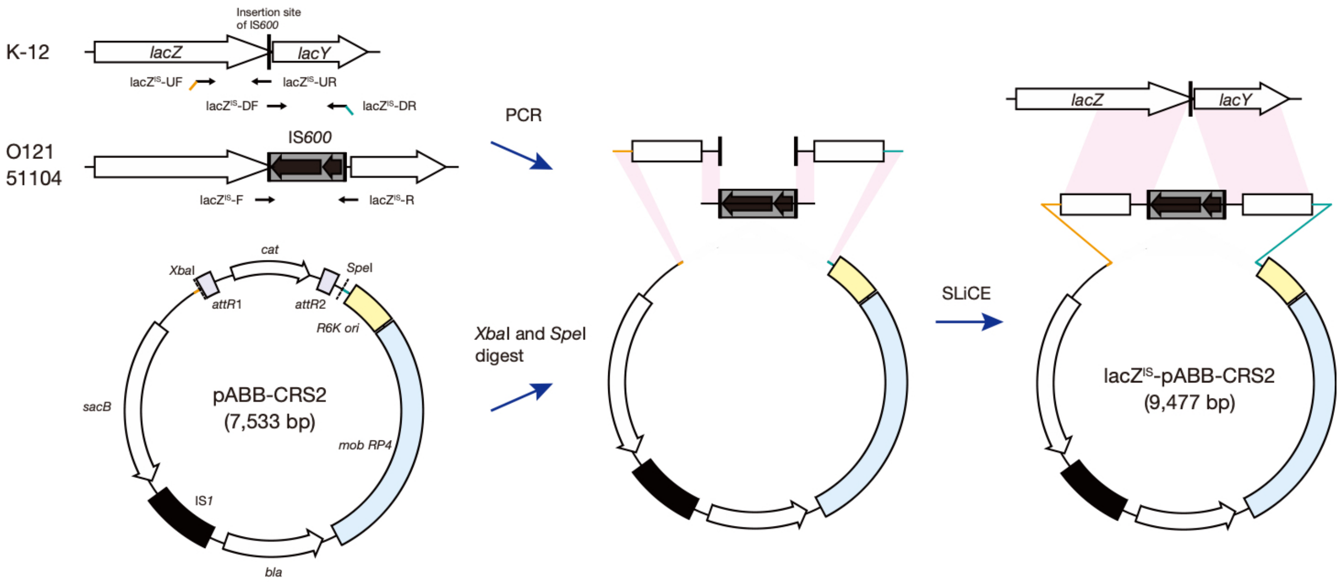

FIG S4. Schematic representation of the construction process of lacZ^{IS}-pABB-CRS2. The regions used for homologous recombination in the SLiCE reaction or in a K-12 cell are indicated in pink.

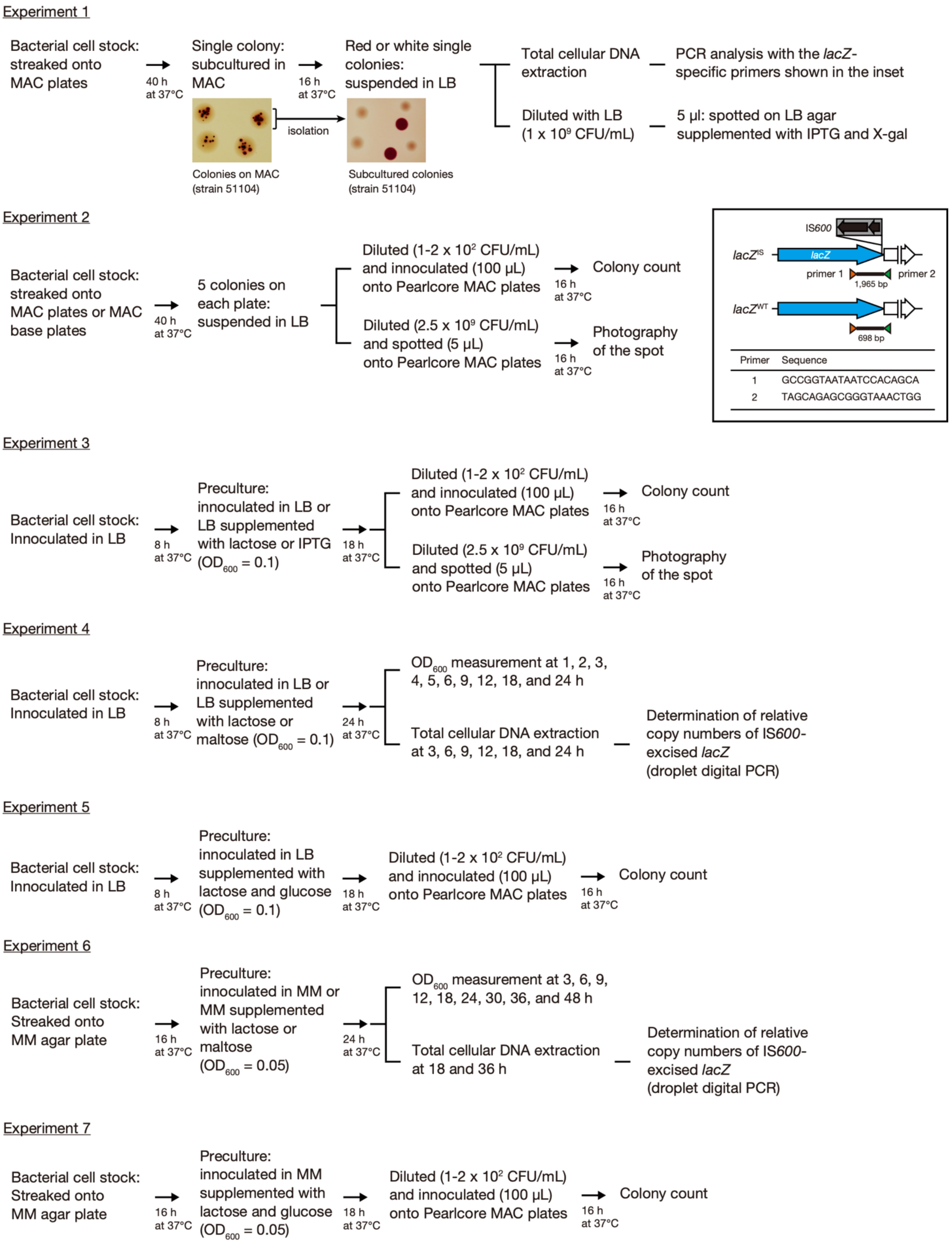

FIG S5. Seven experiments were performed to analyze lactose fermentation, bacterial growth, and the *lacZ* copy number.

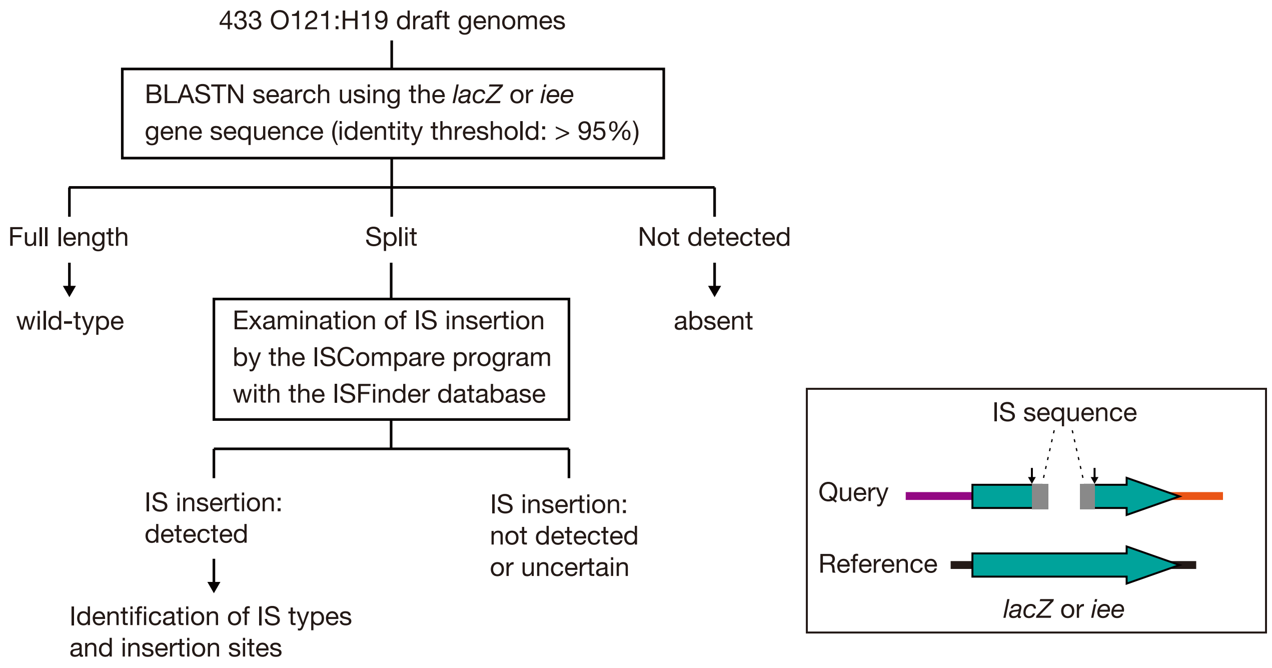

FIG S6. The procedure to examine IS insertion into *lacZ* and *iee*. Draft genomes of O121:H19 (n = 433) were searched by BLASTN using the *lacZ* and *iee* sequences of strain SE14002 (Accession No. AP024473; SE14002_0329 and SE14002_1200, respectively) and the *iee* sequence of strain 51004 (Accession No. AP024471; EC51104_3767) as queries. The nucleotide sequence identity and coverage between the two *iee* genes were 97.6% and 99.1%, respectively. When a split gene was detected, the presence and type of IS insertion were determined by ISCompare (1) with the ISFinder database (2) downloaded from the following site: https://github.com/thanhleviet/ISfinder-sequences (accessed on Oct. 2020). As shown in the inset, ISCompare searched IS sequences in the ends of contigs/scaffolds containing the sequences of split *lacZ* and *iee* genes by comparing them with the reference sequence. Based on the results of a BLASTN search of the detected IS sequence in the ISFinder database, the IS name and its boundaries (indicated by arrows) were determined. As the ISFinder database includes highly similar ISs (>95% identity) under different names, such as IS*600*/IS*Sd1*/IS*Sfl10* and IS*1203*/IS*629*/IS*3411*/IS*1203E*, when such ISs were detected, the representative one IS (for example, IS*600* or IS*1203*) was assigned as the inserted IS. In 11 cases, because IS was identified in one contig/scaffold (see Table S1 in detail), presumed insert positions were assigned to these ISs. In strain FWSEC0155, the *iee* gene was split, but no IS sequences were found.

References

1. Mogro EG, Ambrosis NM, Lozano MJ. 2021. Easy identification of insertion sequence mobilization events in related bacterial strains with ISCompare. *G3* 11:jkab181.
2. Siguier P, Perochon J, Lestrade L, Mahillon J, Chandler M. 2006. ISfinder: the reference centre for bacterial insertion sequences. *Nucleic Acids Res* 34:D32-6.
